# Retinal vessel architecture in retinopathy of prematurity and healthy controls using swept‐source optical coherence tomography angiography

**DOI:** 10.1111/aos.14557

**Published:** 2020-08-04

**Authors:** Sandra Rezar‐Dreindl, Katharina Eibenberger, Reinhard Told, Thomas Neumayer, Irene Steiner, Stefan Sacu, Ursula Schmidt‐Erfurth, Eva Stifter

**Affiliations:** ^1^ Department of Ophthalmology and Optometry Medical University of Vienna Vienna Austria; ^2^ Section for Medical Statistics Center for Medical Statistics, Informatics, and Intelligent Systems (CeMSIIS) Medical University of Vienna Vienna Austria

**Keywords:** choroidal vascular flow area, foveal avascular zone, retinopathy of prematurity, swept‐source optical coherence tomography angiography, vessel density

## Abstract

**Purpose:**

To determine microvascular changes in children with a history of retinopathy of prematurity (ROP) and in a control group of full‐term children.

**Methods:**

In a cross‐sectional study, 30 eyes of 15 children aged 6–8 years with a history of ROP were evaluated with swept‐source optical coherence tomography angiography (SS‐OCTA). Twenty‐eight eyes of 22 age‐matched full‐term children served as a healthy control group. The foveal avascular zone (FAZ), vessel density (VD) and choroidal vascular flow area (VFA) were evaluated on OCTA and correlated with central retinal thickness (CRT), visual acuity (VA), birth weight (BW), gestational age (GA) and ROP stages.

**Results:**

Twenty‐two eyes of 14 children with a history of ROP (stage 1–3) and 25 eyes of 19 full‐term children were available for evaluation. In the ROP group, the gestational age was 27 ± 2 weeks and birth weight was 781 ± 164 g. In the ROP group, CRT was higher in the central ETDRS segment (mean difference [95% CI]: 32.8 µm [18.7; 47.0], p = 0.0002) compared to the controls. Smaller mean FAZ area (−0.12 [−0.19; −0.04], p = 0.004) and perimeter (−662 [−1228; −96], p = 0.03) was found in comparison to the control group. An oval shape of the FAZ was observed among patients with a history of ROP. The mean central VD of the superficial plexus was 28 ± 8/23 ± 8% and of the deep plexus 7 ± 7/3 ± 5% (ROP group/control group; p > 0.05). No statistically significant difference was found regarding the choroidal VFA. Only weak correlation of FAZ and VD with function was observed.

**Conclusions:**

Swept‐source optical coherence tomography angiography imaging revealed significant microvascular anomalies in children with a history of ROP indicating disturbance of early morphological development of the central retina.

## Introduction

Vascularization of the retina depends greatly on oxygen levels and starts around 14–16 weeks of gestation in the posterior superficial retina. The expression of anti‐angiogenic factors leads to the development of the foveal region resulting in the formation of a foveal avascular zone (FAZ), which takes place between 24 and 27 weeks of gestation. Preterm birth may affect the development of the fovea and the vascular plexus due to alterations in retinal oxygenation and expression of factors involved in vascularization (Yanni et al. 2012).

Retinopathy of prematurity (ROP) occurs in children born preterm before the retina is fully vascularized. The pathophysiology of ROP is characterized by a phase of vaso‐obliteration triggered by hyperoxia in association with decreased levels of vascular endothelial growth factor (VEGF) and insulin‐like growth factor 1 (IGF‐1). The vaso‐obliterative phase is followed by a phase of increased VEGF levels. VEGF increase is triggered by retinal hypoxia and can lead to abnormal vessel growth at the interface between the vascularized and nonvascularized retina (Salvin et al. 2010; Selvam et al. 2018). Studies using optical coherence tomography (OCT) have described retinal changes and changes in the foveal morphology in children born preterm and in children with a history of ROP. Compared with full‐term children born >37 weeks of gestation, children born preterm showed a shallower foveal depression, persisting inner retinal layers, a thinner retina and absence of photoreceptor sublayers (Park & Oh 2012; Lee et al. 2018). Furthermore, preterm children with a history of ROP have a higher incidence of ocular changes including a high refractive error, strabismus and glaucoma (Spaide et al. 2015). Beside this, children born preterm are at higher risk for impairment of the visual, cognitive or motor functions. Previous studies demonstrated visual impairment to be associated with neurological deficits in children born preterm and the risk increased in children with a very low birth weight due to the occurrence of complications including intraventricular haemorrhage (O'Connor et al. 2002; Lennartsson et al. 2014).

Using optical coherence tomography angiography (OCTA), which is a noninvasive imaging system for the visualization of the retinal vasculature, the different retinal vascular plexus can be viewed allowing precise localization of a pathology within the retina and choroid (Lee et al. 2018). Optical coherence tomography angiography (OCTA) has been shown to provide reliable images of microvascular abnormalities that can be linked to changes in visual acuity in various retinal pathologies such as age‐related macular degeneration, diabetic retinopathy and retinal vein occlusion (Jia et al. 2012; Shah et al. 2016). For ROP with or without treatment, a few studies using spectral‐domain OCTA (SD‐OCTA) (Falavarjani et al. 2017; Chen et al. 2019; Miki et al. 2019; Nonobe et al. 2019; Takagi et al. 2019) and one study using swept‐source optical coherence tomography angiography (SS‐OCTA) (Bowl et al. 2018) reported an absence of or a smaller FAZ and varying changes in vessel density.

So only one study has used SS‐OCTA, which to date allows retinal imaging with the highest resolution of structural and vascular features available, to evaluate the microvascular abnormalities in children with a history of ROP and full‐term children. Furthermore, limited information exists about the retinal vasculature in children with a history of ROP in different stages. Information about the microvascular changes will help to gain more insight into disease pathomechanisms and potentially allow drawing conclusions on the visual development of these children.

## Methods

The study adhered to the tenets of the Declaration of Helsinki and was approved by the local ethics committee of the Medical University of Vienna. We included 30 eyes of 15 children aged 6–8 years with a history of ROP (ROP 1–3) in this cross‐sectional study. Children with ROP stage 1–2 without treatment and children with ROP 3 receiving laser coagulation of the retina were included. Twenty‐eight eyes of 22 age‐matched full‐term children with a gestational age over 38 weeks served as a healthy control group. All children underwent a full ophthalmological examination including visual acuity (VA), slit lamp examination and funduscopy. In addition, the weight at birth (BW), gestational age (GA) and data about a previous laser treatment of the retina was recorded and analysed.

Exclusion criteria comprised children with high refractive errors of >±6dpt, glaucoma, uveitis, ocular trauma, other retinal diseases or cataract. Eight eyes of the ROP group and three eyes of the control group had to be excluded from analysis due to insufficient image quality. Hence, final sample size was 14 children (22 eyes) in the ROP group and 19 children (25 eyes) in the control group.

### Swept‐source optical coherence tomography angiography

All children were examined after pupil dilatation (Tropicamide, Mydriaticum Agepha, Austria) using the PlexElite 9000 (Carl Zeiss Meditec, Dublin, CA, USA) swept‐source OCTA device (SS‐OCTA). The SS‐OCTA device uses a 1060‐nm swept source with an A‐scan rate of 100 000 and an axial resolution of 6.3 µm. A 3 × 3 mm volumetric flow scan centred on the fovea containing 300 A‐scans per 300 B‐scans was recorded for each eye. Immediate assessment of the scan quality allowed for repeating SS‐OCTA scanning if the image quality was poor due to, for example, motion artefacts. Built‐in online motion correction to minimize artefacts was used for each scan automatically. The automated layer segmentation displays the predefined vascular plexuses. These are the superficial and deep retinal capillary plexus, the outer retina to choriocapillaris (ORCC) and the choroidal capillary (CC) layer in orthogonal view. Layer segmentation was corrected manually whenever necessary. The corresponding structural B‐scans were used to guide placement of the segmentation lines.

### Evaluation of swept‐source optical coherence tomography angiography‐based variables

Swept‐source optical coherence tomography angiography (SS‐OCTA) images were exported from the device. The superficial capillary plexus was used for manual delineation of the FAZ, allowing calculation of the FAZ area, perimeter and circularity. This was done using the freehand selection tool of Fiji 1.50e (Image J, National Institutes of Health, Bethesda, MD, USA) (Schindelin et al. 2012). The perimeter was defined as the length of the outside boundary of the FAZ. The circularity of the FAZ was measured and a value of 1.0 indicated a perfect circle. As the value approaches 0.0, it indicates an increasingly elongated shape.

The vessel density (VD) was evaluated online for the superficial and deep vascular plexus as well as the choriocapillaris, using the automated segmentation. The VD was evaluated for the inner circle (segments 1–5) of the ETRDS grid, fitting the 3 × 3 mm SS‐OCTA images and allowing evaluations of the central millimetre, the superior and inferior as well as the nasal and temporal ETDRS fields. In addition, the SS‐OCTA images obtained were used to determine the average retinal thickness. Retinal thickness was analysed on 3 × 3 mm scans in the different segments of the ETDRS grid and was analysed from internal limiting membrane to Bruch membrane. The vascular flow area (VFA) of the choriocapillaris was determined using Fiji 1.50e (Image J, National Institutes of Health) as described (Nicolò et al. 2017). The VFA represents the percentage flow area in relation to the total area. In short, the Otsu threshold was applied to the choriocapillaris slab, which was consequently binarized to determine the number of black/white pixels, allowing the calculation of the VFA.

### Statistical analyses and outcome measures

For quantitative variables, mean ± SD are reported or median (min; max). Qualitative variables are reported as absolute and relative frequency. The primary endpoints of the study are superficial and deep vascular plexus. Secondary endpoints were retinal thickness, FAZ (perimeter, area and circularity), and the VFA of the choriocapillaris. As some patients were measured on both eyes, group comparison (children born preterm with history of ROP versus full‐term children without history of ROP) was done by mixed models (SAS Proc mixed). We included patient as a random factor in the model to consider that measurements between two eyes of the same child may be stronger correlated than measurements between two eyes of different children. As previous studies reported negative association between central retinal thickness with FAZ and VD, adjustment for retinal thickness (within the respective analysed ETDRS Segment for VD deep and superficial, or for FAZ and VFA within the central ETDRS Segment) was done. For the variables VD deep, VD superficial and retinal thickness, mixed models were calculated for each ETDRS Segment separately. An estimate with 95% confidence interval for the mean group difference and the p‐value (H0: equal means in ROP and control group), as well as the number of valid observations are reported. In addition, within the ROP group, FAZ (area, perimeter, circularity) and VD (deep, superficial) were correlated with central retinal thickness, visual acuity, birth weight, gestational age and ROP stage. Graphical visualization of group differences was done by boxplots. Spearman correlation coefficients with 95% confidence interval are reported (R‐package DescTools, R‐function SpearmanRho). As the assumption of independent observations is not met (for some patients, both eyes are measured), correlation analyses were done for the right and left eye separately. Statistical analyses were conducted with R 3.6.0 under RStudio 1.0.143 (The R Foundation for Statistical Computing, Vienna, Austria) and SAS 9.4 (SAS Institute, Cary, NC, USA). p‐Values <0.05 were considered statistically significant. As the analyses are exploratory, no adjustment for multiple testing was done.

## Results

We included 22 eyes of 14 children with a history of ROP (ROP 1–3) and 25 eyes of 19 full‐term children in our analysis. The mean age of the children was 7 ± 1 and 8 ± 1 years (ROP/control group; p = 0.3), respectively. In the ROP group 8/6 (57/43%) were male/female and in the control group 10/9 (53/47%). All children had symmetric ROP stage in both eyes. Three children had ROP 1 and six children had ROP 2, which resolved spontaneously without treatment. Five children had a history of ROP 3 receiving laser coagulation of the retina. All of the patients with ROP stage 3 showed plus disease. 43/32% of the children in the ROP/control group were myopic with myopia of less than −5 dioptres (dpt) and 36/53% hyperopic with less than +5dpt. In the ROP group, the mean gestational age was 27 ± 2 (range 23 + 0‐29 + 2) and the mean weight at birth was 781 ± 164 g (range 470–1062 g). Median visual acuity (min; max) was 1 (0.4; 1) LEA in children with a history of ROP and 1 (0.7; 1.6) LEA in the control group. Visual acuity significantly differed between the ROP and control group (mean difference [95% CI] ROP versus control: −0.18 [−0.34; −0.02], p = 0.034). The characteristics of the patients with a history of ROP are shown in Table 1.

**Table 1:** Characteristics of the patients with history of ROP (*n* = 14).

	Mean ± SD	
Age (years)	7.3 ± 1.4	
Gestational age (weeks)	27 ± 2	
Birth weight (g)	781 ± 164	
	**Number of patients**	**Percentage**
Female	6	43
Male	8	57
ROP		
ROP 1	3	21
ROP 2	6	43
ROP 3	5	36
Treatment		
ROP with no treatment	9	64
ROP with previous laser coagulation	5	36

ROP = retinopathy of prematurity; SD = standard deviation.

### Optical coherence tomography angiography

**Fig. 1:**
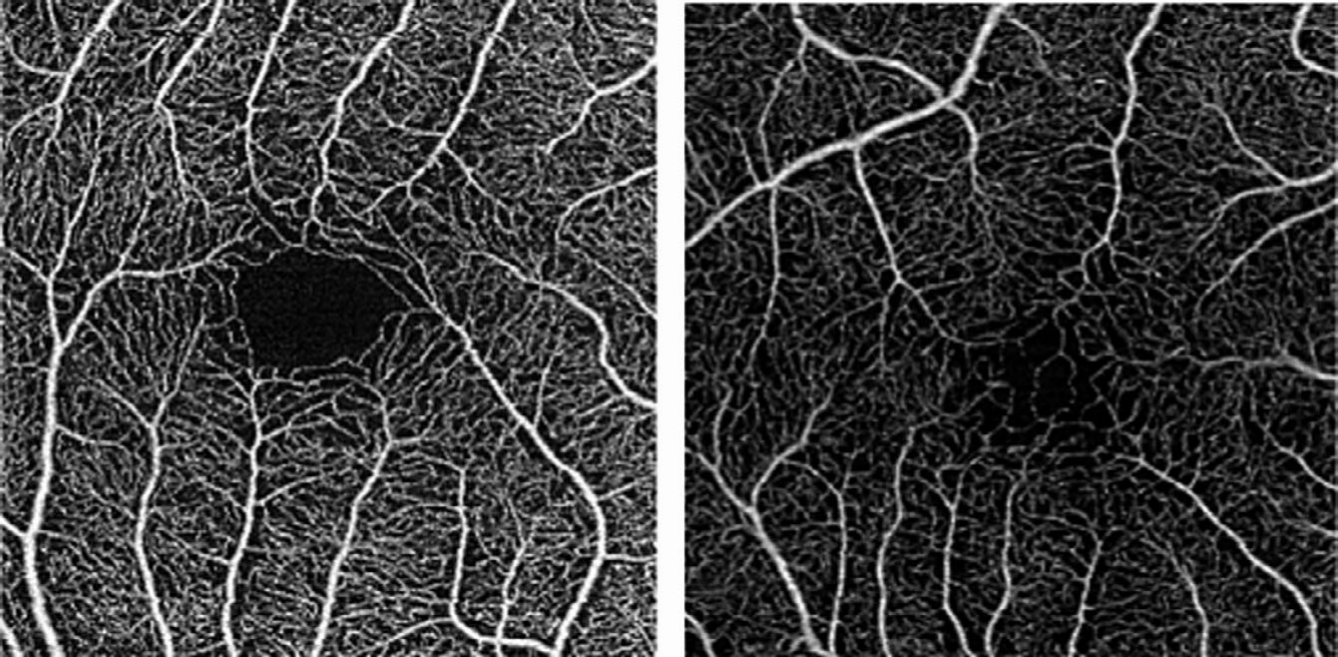
Top left*:* Swept‐source optical coherence tomography angiography (SS‐OCTA) image of an 8‐year‐old full‐term child with a foveal avascular zone (FAZ) of 0.3 mm and a perimeter of 2.6 mm. LEA visual acuity 1.0. Top right: SS‐OCTA image of a 7‐year‐old child with a history of retinopathy of prematurity (ROP) stage 1 showing a small superficial FAZ area of 0.07 mm with a perimeter of 1.4 mm. LEA visual acuity 0.6. Bottom lefts: SS‐OCTA of a 6‐year‐old child with a history of retinopathy of prematurity stage 2 showing a small superficial FAZ area of 0.09 mm with a perimeter of 1.3 mm. LEA visual acuity 0.5. Bottom right: SS‐OCTA of an 8‐year‐old child with a history of retinopathy of prematurity stage 3 showing a small superficial FAZ area of 0.01 mm with a perimeter of 0.6 mm. LEA visual acuity 0.6.

**Fig. 2:**
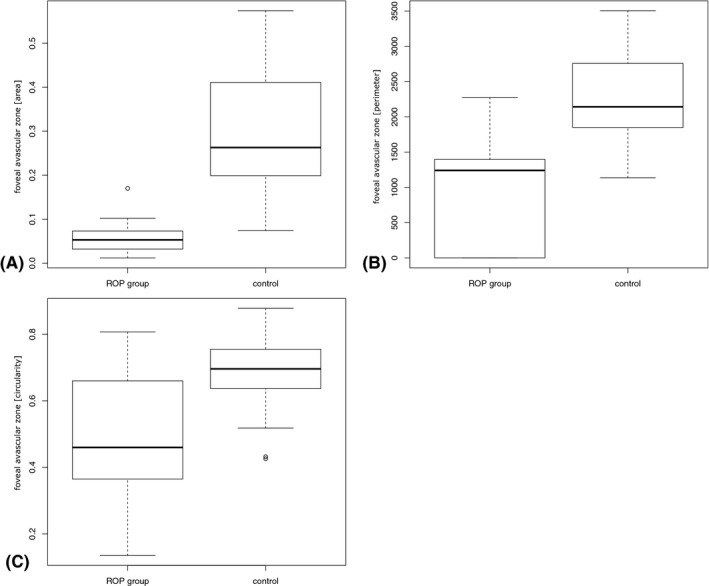
Foveal avascular zone (A) area (B) perimeter and (C) circularity.

**Fig. 3:**
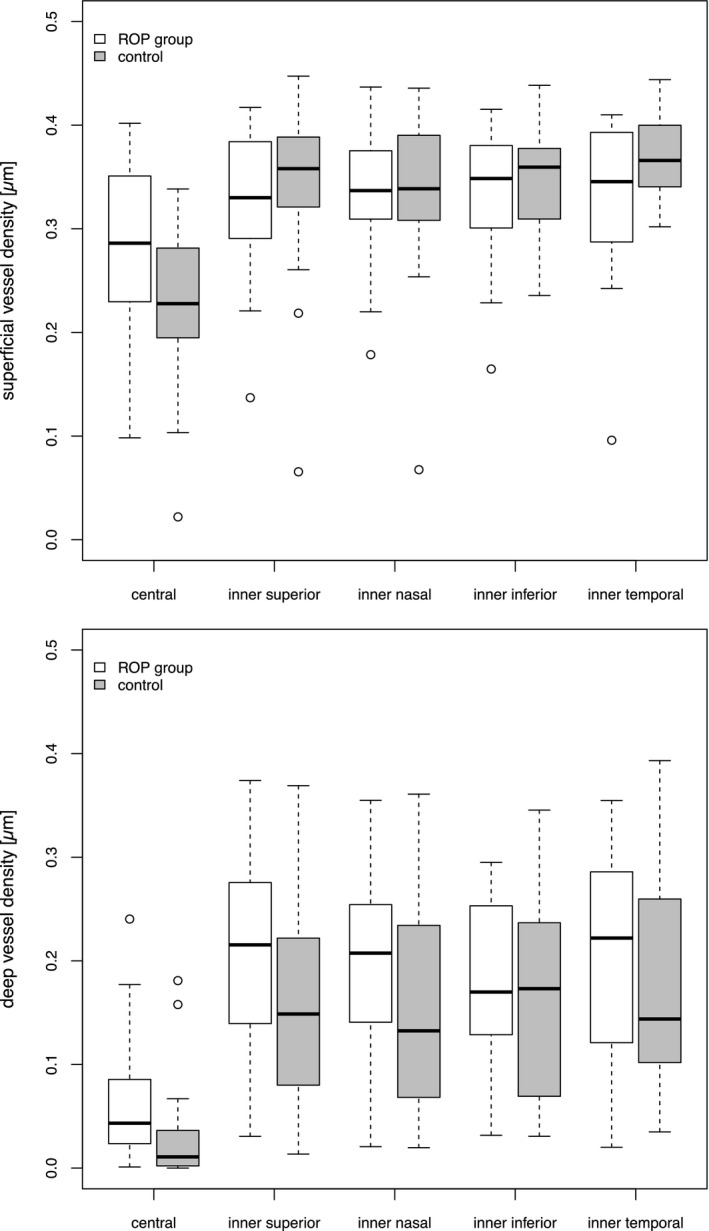
Vessel density of the superficial and deep vascular plexus in the segments of the ETDRS grid of children with a history of retinopathy of prematurity and of the control group.

Table 2 shows a summary of the OCTA measurements in the ROP and the control group. Differences were found between the ROP and control group regarding FAZ area, perimeter and circularity (Figures 1 and 2). The FAZ area was smaller with a reduced perimeter and a more ellipsoid shape (area: mean difference [95% CI]: −0.116 [−0.189; −0.044], p = 0.004; perimeter: −662 [−1228; −96], p = 0.03; circularity: −0.155 [−0.303; −0.007], p = 0.04). No statistically significant difference was found regarding FAZ area (0.01 [−0.036; 0.056], p = 0.63), perimeter (−131 [−1011; 748], p = 0.74) and circularity (−0.030 [−0.195; 0.254], p = 0.77) in children with ROP receiving laser coagulation (*n* = 5) versus ROP 1–2 (*n* = 9) with spontaneous resolution. Within our analysis no patient received anti‐VEGF treatment. Vessel density (VD) was evaluated for the superficial and the deep vascular plexus in the five segments of the ETDRS grid centred on the fovea. No statistically significant difference in VD was found between the ROP and control group with and without adjustment for retinal thickness. The mean central VD of the superficial plexus was 28 ± 8/23 ± 8% (ROP group/control group; 2.6% [−5.6; 10.7], p = 0.5) and of the deep vascular plexus was 7 ± 7/3 ± 5% (ROP group/control group; 4.7% [−0.96; 10.3], p = 0.09). Figure 3 shows the VD of the superficial and deep vascular plexus in the five ETDRS segments. Choroidal VFA was 51.4 ± 2.6% in children with history of ROP and 51.0 ± 0.6% in the control group (−0.019% [−23.6%; 23.5%], p = 1).

**Table 2:** Optical coherence tomography angiography (OCTA) measurements in children with a history of ROP and controls.

	ROP group	Control group	p‐Value
Foveal avascular zone			
Area (mm)	0.1 ± 0.04	0.3 ± 0.1	0.004
Perimeter (µm)	1036 ± 683	2257 ± 620	0.03
Circularity (a.u.)	0.5 ± 0.2	0.7 ± 0.1	0.04
Vessel density			
Superficial vascular plexus (%)			
Central	28 ± 8	23 ± 8	0.5
Inner superior	33 ± 7	34 ± 8	0.5
Inner nasal	33 ± 7	34 ± 8	0.6
Inner inferior	33 ± 7	35 ± 5	0.4
Inner temporal	33 ± 8	37 ± 5	0.1
Deep vascular plexus (%)			
Central	7 ± 7	3 ± 5	0.09
Inner superior	21 ± 1	16 ± 10	0.3
Inner nasal	20 ± 9	15 ± 10	0.3
Inner inferior	18 ± 8	17 ± 10	1
Inner temporal	20 ± 1	19 ± 11	0.9
Choroidal vascular flow area			
Vascular flow rate (%)	51 ± 3	51 ± 6	1

a.u = arbitrary units, ROP = retinopathy of prematurity.

Data presented as mean ± standard deviation.

### Central retinal thickness

**Fig. 4:**
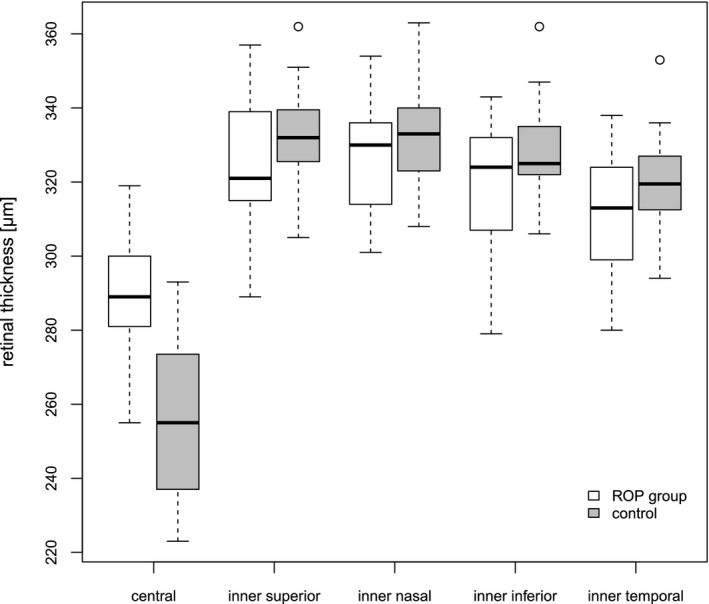
Retinal thickness in the segments of the ETDRS grid of children with a history of retinopathy of prematurity and of the control group.

Central retinal thickness (CRT) was measured on OCT in the five inner segments of the ETDRS grid. Average CRT (ROP group/control group) was 290 ± 17/256 ± 19 µm (central, mean group difference [95% CI]: 32 µm [19; 47], p = 0.0002), 326 ± 15/333 ± 12 µm (inner nasal; −8 µm [−19; 3], p = 0.1), 325 ± 18/333 ± 13 µm (inner superior; −9 µm [−21; 3], p = 0.1), 311 ± 16/320 ± 12 µm (inner temporal; −10 µm [−21; 0.7], p = 0.07), 319 ± 18/329 ± 12 µm (inner inferior; −11 µm [−23; 0.5], 0.06). Figure 4 shows the CRT of patients with history of ROP and patients in the control group.

### Correlations

Weak correlations could be observed between retinal thickness and FAZ or VD, respectively. The FAZ area negatively correlated with retinal thickness (spearman *r* = −0.57 [−0.89; 0.16], *n* = 9 and −0.59 [−0.87; −0.02], *n* = 12 for right and left eye, respectively). Furthermore, the vessel density of the superficial and deep vascular plexus within ETDRS Segment 1 showed weak correlation with BW, GA and visual acuity, as well as with the different ROP stages. Note that due to the low sample size, confidence intervals are wide.

## Discussion

Preterm birth and ROP are associated with changes in retinal oxygen levels and may lead to alterations in the development of the retinal vascular plexus, fovea and FAZ. Optical coherence tomography has shown retinal morphology to be different in children born preterm. Spectral‐domain OCT images showed that a foveal depression is broad and shallow in preterm infants with a history of ROP (Villegas et al. 2014).

In our analysis, we used SS‐OCTA to compare the retinal vessel architecture in children born preterm with a history of retinopathy of prematurity (ROP 1–3) and full‐term children. Our data showed a smaller FAZ area together with reduced perimeter and an ellipsoid shape among patients with a history of ROP stage I–III. Previous studies of preterm children with a history of ROP using spectral‐domain OCTA have reported the FAZ absent or reduced. (Falavarjani et al. 2017; Bowl et al. 2018; Chen et al. 2019; Miki et al. 2019; Nonobe et al. 2019; Takagi et al. 2019). Spectral‐domain OCTA has been used to evaluate the FAZ in children with a history of different stages of ROP and following laser coagulation of the retina or anti‐VEGF treatment. A smaller FAZ area compared with a healthy control group was reported among children who had received anti‐VEGF treatment (Chen et al. 2019) and in children with a history of ROP (1–2) or of ROP 3, who received laser coagulation (Falavarjani et al. 2017; Miki et al. 2019; Nonobe et al. 2019; Takagi et al. 2019). In our analysis we measured the area, perimeter and circularity of the FAZ and found differences between children with a history of ROP and healthy full‐term children. These results accord with histopathological findings, which showed that by 37 weeks of gestation the FAZ is a complete circle and decreases in size until 41 gestational weeks, but the fovea finally reaches maturity after 15–45 months of age (Hendrickson & Yuodelis 1984; Provis & Hendrickson 2008).

However, only weak correlations of the FAZ area, perimeter and circularity with visual acuity were observed within the ROP group (Spearman correlation coefficients ranging from −0.18 to 0.09). Some previous studies have also failed to detect any correlation between the area of the FAZ and visual acuity. It was demonstrated that in the absence of a foveal pit cone specialization is present and no disturbance of visual acuity resulted. Accordingly, other factors unrelated to the morphological development of the fovea and FAZ may be causative in cases of reduced visual development in preterm children with a history of ROP. Our patient population mainly comprised patients showing good visual development with a median (min; max) visual acuity of 1 (0.4; 1) LEA in the ROP group. More studies are needed to evaluate those patients with strongly reduced visual acuity development to investigate the factors and morphological changes leading to worse visual acuity. In addition, in 1.5% of healthy eyes with normal visual acuity an absent FAZ with macular‐foveal capillaries was reported (Yokoyama et al. 2018), and in those patients, an abnormal foveal mesopic and scotopic function was detected without association to an ocular disease (Pilotto et al. 2019). Additional visual function tests should be done in order to further investigate the visual function in those patients. Furthermore, in our study, we detected no relevant correlation regarding the different ROP stages (1–3) and no significant difference between patients receiving laser coagulation and patients with spontaneous resolution of ROP. In a previous study, the FAZ was found to be absent in 60% of children with ROP with a history of laser therapy and in only 33% of children with preterm birth without laser therapy (Falavarjani et al. 2017). It is possible that the different finding may be attributable to the small sample size or to the fact that in the previous study patients with history of prematurity were compared to children receiving laser coagulation. No comparison between the different ROP stages was one. However, further studies are needed to investigate differences between the different stages of ROP and in patients receiving treatment.

Furthermore, it was shown before that the central retinal thickness was correlated with the FAZ and the VD. In our study, retinal thickness showed a negative correlation with the area of the FAZ. Developmental variations of the FAZ are likely associated with inner retinal layers changes at the foveal centre. Also, a relation between a thicker retina and an incomplete formation of the foveal depression has been assumed to be associated with the severity of ROP and previous laser coagulation of the retina. Our data also showed a negative association between the FAZ and retinal thickness. In addition, a previous study using spectral‐domain OCTA reported the mean CRT to be significantly thicker in an ROP group than in a control group (228 and 189 µm, p < 0.01) (Sjöstrand et al. 2017; Lutty & McLeod 2018; Takagi et al. 2019), which accords with our findings.

The superficial vascular plexus begins to develop at around 21 weeks of gestation and the deep vascular plexus around 25–26 weeks of gestation (Hughes et al. 2000). Histopathological studies have shown that during maturation the macular is never vascularized, with only cones present in this area (Provis & Hendrickson 2008). The researches attributed the formation of an avascular fovea to an elevated expression of pigment epithelium‐derived factor and brain natriuretic peptide precursor B, which they detected in the foveal area. In our analysis, vessel density of the superficial and deep vascular plexus in the central ETDRS segment of children with a history of ROP showed the greatest difference compared with healthy full‐term children. The VD of the superficial and deep plexus was on average higher in the ROP group than in the control group (28/23% and 7/3%; p = 0.5 and p = 0.09), however, the difference failed to reach statistical significance. Previous studies have reported a higher VD in the superficial plexus varying between 39% and 42% in children born preterm with/without history of ROP and with/without treatment (anti‐VEGF/laser coagulation) and between 32% and 33% in the control group. One study showed a mean VD of the deep vascular plexus of 35% in the ROP and 28% in the control group. In these studies, vessel density was only evaluated in the central foveal VD or the superficial plexus (Falavarjani et al. 2017; Chen et al. 2019). In a study using SS‐OCTA, the superficial and deep vascular plexus were reported to be fused together in the foveal centre with a single vascular network developed in eyes with an absent FAZ. Therefore, only the VD of the superficial plexus was reported (Falavarjani et al. 2017). Other researchers have defined VD as the percentage of the area occupied by the vessels in a particular area in mm^2^/mm^2^ and reported no difference in the median VD of the central ETDRS segment between an ROP and control group. However, differences were reported for the median VD in the average of the ETDRS segments 2–5 (Nonobe et al. 2019). We found no statistically significant difference in VD of the superficial and deep vascular plexus in either segment of the ETDRS grid with and without adjustment for retinal thickness. Spectral‐domain and SS‐OCTA is used for the evaluation of different retinal pathologies including the detection of choroidal neovascularization and in central serous chorioretinopathy. SD‐OCTA uses a light source with a wavelength of 840 nm, whereas SS‐OCTA uses a wavelength of 1050 nm. The use of a longer wavelength enables a better penetration through the retinal pigment epithelium and a stronger signal from the deeper structures. This advantage of a better visualization of the deeper layers has been shown in studies evaluating choroidal neovascularization. With SD‐OCTA, imaging detection of choroidal neovascularization under the RPE is limited while SS‐OCTA is able to detect vascular patterns associated with the choroidal neovascularization more reliably (Told et al. 2016). Furthermore, evaluations comparing the SD and SS‐OCTA showed that the advantages of SS‐OCTA imaging include a faster scanning speed leading to a denser scan pattern, which captures larger scan areas (Novais et al. 2016; Told et al. 2016).For children with ROP differences exist regarding the definition of the VD and among patient cohorts with varying changes in VD among ROP and control groups. Consequently, investigations focusing on changes in the VD at different stages of ROP and after treatment are necessary to further explore the vascularization of the superficial and deep plexus. Possible confounders including the retinal thickness need to be detected and taken into account when interpreting the VD.

Choroidal development starts before the development of the retinal vasculature. After birth continuous maturation of the choroid was shown (Hardy et al. 1996). Previous studies among preterm infants with ROP reported thinner choroidal thickness which was more pronounced with severity of ROP and which was associated with worse visual acuity (Wu et al. 2013; Erol et al. 2016). Involution of the choroidal vasculature may lead to decreased oxygen delivery to the outer retina. With the usage of SS‐OCTA the choriocapillaris can be reliably imaged in vivo. Significant thinner choroidal vascularity index, which represented the proportion of luminal areas to the total subfoveal choroidal area, was shown for children with ROP (Lavric et al. 2019). Among our cohort of patients with ROP the VFA of the choriocapillaris was measured reflecting the percentage flow area in relation to the total area. No difference was observed regarding the VFA of the choriocapillaris. However, our study comprised patients with different ROP stages with or without previous treatment and larger studies are needed to evaluate the differences between children with or without previous treatment. Limited investigation of the choroid and ROP exists and further studies are needed to determine the impact of the choroid on ROP.

Our study has some limitations that need to be mentioned. The sample size of the study was small and it lacked a control group of preterm infants without a history of retinopathy of prematurity. We reported the 95% confidence limits of the mean group differences in the manuscript and thus provide information about the uncertainty of the estimation due to the small sample size. However, the recruitment of patients in this special cohort is also limited by the fact that there are low numbers of patients available. The morphological changes observed in our study may have resulted from ROP or prematurity alone. However, using SS‐OCTA we could demonstrate microvascular anomalies including a smaller FAZ together with a reduced perimeter and more ellipsoid shape in children with a history of ROP compared with a healthy control group. The amount of VD in the superficial and deep vascular plexus and the vascular flow rate was reported among patients with a history of ROP at different stages. No relevant correlation of these finding with the severity of disease and the functional outcome was observed. Further investigations are needed to explore the microvasculature in preterm children to gain more insight into disease pathomechanism and functional outcome.
